# COVID-19 pandemic

**DOI:** 10.5935/0103-507X.20200001

**Published:** 2020

**Authors:** Marcelo Moock, Patrícia Machado Veiga de Carvalho Mello

**Affiliations:** 1 Hospital Regional de São José dos Campos - São José dos Campos (SP), Brazil.; 2 Hospital de Terapia Intensiva - Teresina (PI), Brazil.

Once again, a disease advances and worries everyone. Unlike other epidemics and thanks to advances in science, this flu was considered distinct, its etiological agent isolated and identified, and new detection tests were developed in record time. So far there are no effective vaccines or active antiviral drugs for SARS-2, the coronavirus responsible for the so-called COVID-19 flu.

The pandemic is spreading from Asia to the West. We have qualified epidemiological and technical information, through scientific reports from China and other countries that have been published in the main categorized scientific journals. There is an effort by the World Health Organization (WHO) and the scientific community, in general, to order and consolidate this information, which is essential for the guidance of health professionals and managers in the construction of safe and effective strategies for coping with the pandemic.

The *Associação de Medicina Intensiva Brasileira* (AMIB) is contributing to the Crisis Offices and Task Forces installed at different levels of government to instruct these councils with qualified information. Based on the recommendations of these councils, the authorities apply relevant measures to contain or mitigate the spread of the virus.

The assistance battlefront is another relevant focus of AMIB’s effort. Our challenge will be to provide the best possible care to patients, with safety for the professionals in charge of their respective work shifts. Available reports report that there has been occupational contamination and even deaths of infected professionals. In view of this evidence, it is essential to adopt the measures already disclosed by AMIB, available on its website (https://www.amib.org.br), with all rigor. Any breach of the announced protocols is not safe. Adherence to these recommendations will increase the time dedicated to the care of patients and managers should be alert to mobilize other professionals for back-up tasks, thus relieving those who are directly involved in caring for patients. Another responsibility of the Technicians and Managers is to ensure that all recommended personal protective equipment is available at all times. Our strength should not be exposed to additional risks. Whenever possible, young people should be assigned to direct care. Older people can supervise and carry out rear tasks. Visits and the flow of people in the units must be restricted.

The authorities announce the expansion of intensive care services in Brazil to face the pandemic. Qualified personnel may not be available in all locations. AMIB will support expansion and provide remote consultancy to doctors involved in the care. Our goal is to reduce mortality and morbidity. There is a huge amount of knowledge accumulated in the association and it is necessary to expand and make available this important asset at this moment.

Our community is mobilized and, although there is apprehension, natural and desirable for adherence to safety guidelines, the flame that moves us is to take care of everyone and save the majority.

